# Supplementary material for: A Global Screen for Assembly State Changes of the Mitotic Proteome by SEC-SWATH-MS
Source: Cell Syst. 2020 Feb 26;10(2):133–155.e6. doi: 10.1016/j.cels.2020.01.001 (PMC7042714; doi:10.1016/j.cels.2020.01.001)

Q9UBT2 | SAE2_HUMAN | UBA2 SAE2 UBLE1B HRIHFB2115
Monomer MW [kDa]: 71.224 Monomer expected elution fraction: 43

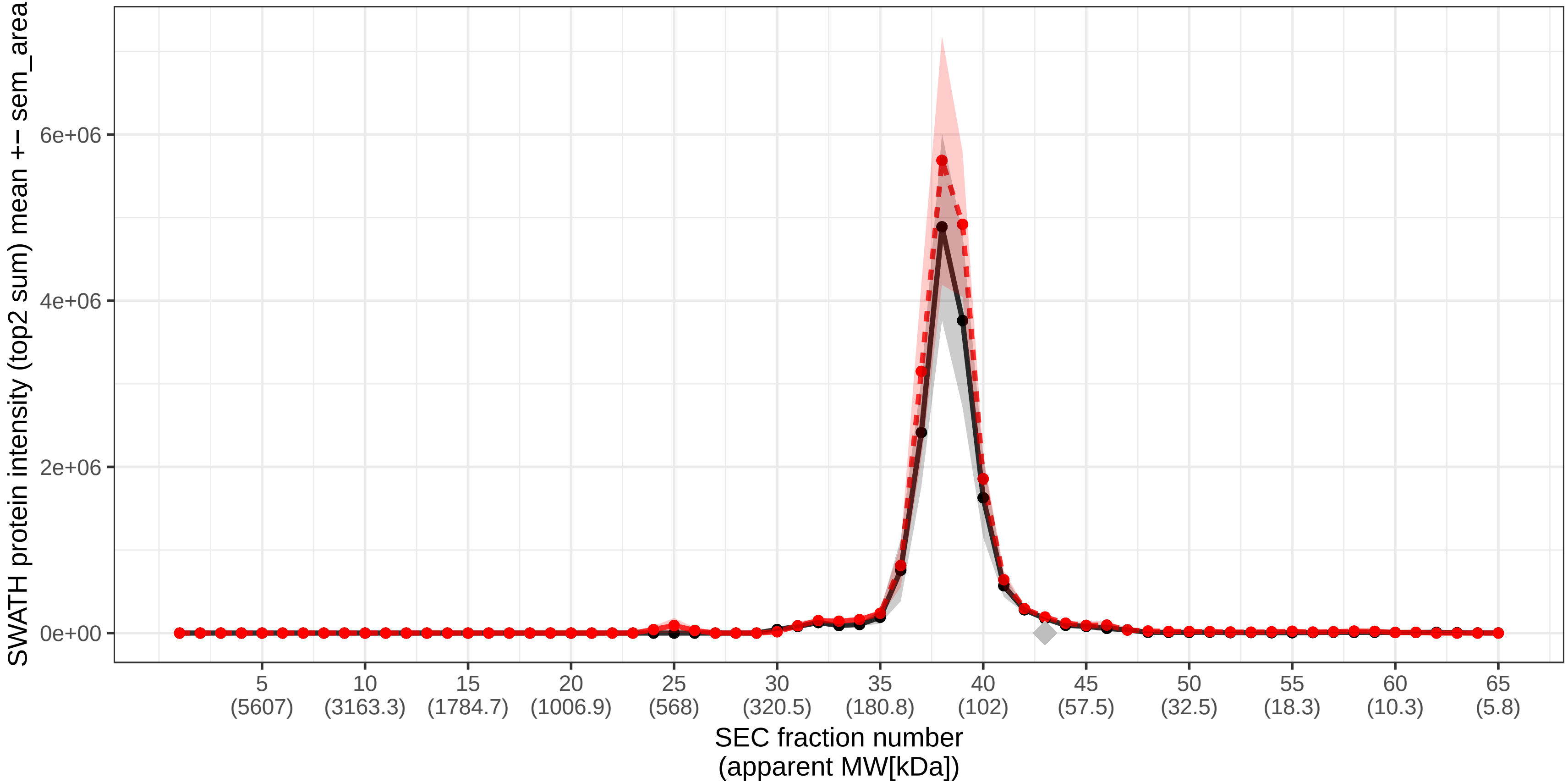

Supplement: Data S1. SEC-SWATH-MS Protein Chromatograms, Related to Figure 1 [file mmc6.zip › SECchrom_Q9UBT2_SAE2_HUMAN_UBA2_SAE2_UBLE1B_HRIHFB2115.pdf]
